# Gouldian Finches (*Chloebia gouldiae*) Increase the Frequency of Head Movements with Increasing Risk at Water-holes but Prolong Interscan Intervals While Drinking: Two Different Strategies?

**DOI:** 10.3390/ani16010087

**Published:** 2025-12-28

**Authors:** Gerhard Hofmann, Claudia Mettke-Hofmann

**Affiliations:** 1 Independent Researcher, Moreton CH46, UK; gerhard@hofmann-photography.de; 2 School of Biological & Environmental Sciences, Liverpool John Moores University, Liverpool, L3 3AF UK

**Keywords:** vigilance, social vigilance, alertness, birds, Estrildidae

## Abstract

Animals scan their environment to detect threats. Such vigilance behaviour is costly, and animals adjust their vigilance to prevailing threats. Waterholes are dangerous places that attract predators, and require heightened vigilance. We investigated how Gouldian finches adjust their vigilance at waterholes by measuring the frequency of head movements and interscan interval while drinking. The frequency of head movements increased with increasing risk perception (a) from perching in the tree to being on the ground and (b) while drinking, with a higher frequency of movements recorded at small waterholes compared to medium and large ones. The latter adds to recent findings that small waterholes are perceived as more dangerous and require further investigation. With a predicted increase in droughts, birds will rely on small waterholes for longer during the dry season which might cause stress. Furthermore, interscan interval varied in terms of interaction with waterhole size and group size, indicating adjustments linked to perceived threat and social effects. Finally, frequency of head movements and interscan intervals were positively correlated. This might reflect different strategies to check the environment, with either a high frequency of head movements when scanning accompanied by long drinking bouts or looking in one direction for a longer time (lower vigilance) but repeating this behaviour at shorter intervals.

## Simple Summary

Animals scan their environment to detect predators. However, being vigilant is costly as animals must interrupt other activities. Here, we investigated vigilance at waterholes, which are dangerous sites as they attract predators. We assessed vigilance during drinking by measuring the frequency of head movements when the birds raised their head above the water surface and also measured the time taking-in water (interscan interval). The frequency of head movements was lower when the birds sat in trees when compared to being on the ground, indicating that the birds felt more vulnerable as they moved closer to the ground. While drinking, the frequency of head movements was highest at small waterholes when compared to larger waterholes, showing that small waterholes are perceived as more dangerous. This is concerning as droughts are predicted to increase. Furthermore, interscan interval was affected by waterhole size and group size in a complicating manner, showing that birds adjust their vigilance levels in response to specific situations. Finally, frequency of head movements and interscan interval were correlated. Individuals with a high frequency of head movements (high vigilance) had longer drinking bouts, whereas individuals with a lower frequency of head movements (lower vigilance) raised their head after shorter drinking intervals, possibly adjusting for the lower frequency of head movements.

## 1. Introduction

Animals keep track of changes in their environment to avoid predation but also to monitor their companions to coordinate movement and avoid aggression by scanning their environment (vigilance; refs. [1,2,3,4,5]). This behaviour is costly as individuals usually must interrupt foraging or other activities to scan the environment [6]. Adjusting vigilance to the prevailing threats is important to balance costs and benefits of vigilance.

Animals can adjust several parameters when vigilant: they can vary the overall amount of time spent being vigilant but also vary the duration of single vigilant bouts, the rate of being vigilant (how often to interrupt other activities to scan) and the length of the intervals between successive vigilance bouts (interscan interval). These parameters have been mainly investigated in a foraging context as vigilance (heads-up) and foraging (head-down) are often in conflict with each other [6,7,8] and allow us to investigate how animals balance costs and benefits. Generally, a higher proportion of overall vigilance [9,10,11,12,13,14] and a higher rate of heads-up [9,14,15] are associated with increasing threat perception [9,11,12,14,15], smaller groups [13] or being at the periphery [10,13]. While both vigilance patterns are associated with higher costs (less time for other activities [6,7,8]), they benefit the individual; for example, a higher rate of vigilance has been linked to better predator detection [16].

Few studies have measured interscan intervals but such studies have found interesting relationships between scan duration and rate of vigilance. Generally, interscan interval decreased with increasing threat (predation: refs. [2,17], noise: ref. [18]). Moreover, individuals that were more exposed to risk of predation (proactive personality [19]; conspicuousness [20], higher pecking rate [16]) had shorter interscan intervals and higher scan rates than individuals less exposed to risks. Sirot and Pays [21] predicted shorter interscan intervals and longer scan durations when arriving in novel environments to assess threats. In Siskins (*Carduelis spinus*), interscan intervals were shorter with no decrease in scan duration under high predation, whereas social competition increased scan duration but did not change the interscan interval [2]. Finally, Turnstones (*Arenaria interpres*) with better body conditions had shorter interscan intervals than birds with poorer body conditions [22].

Additionally, while being vigilant, animals often turn their head in different directions to collect information about different parts in their environment. They can use different strategies by varying the frequency of their head movements [23]. A higher frequency of head movements allows an animal to scan a larger area within a shorter period of time, whereas fewer head movements allow an animal to obtain more detailed information about a threat (predator or rival; ref. [23]). The choice of strategy used can be investigated within and outside foraging contexts. A higher frequency of head movements has been shown to be prevalent in more dangerous situations [24,25], when in smaller groups [26,27] or at the periphery [26]. Therefore, a higher frequency of head movements relates to higher vigilance and has been suggested to result in higher predator detection [25].

A major factor in reducing vigilance and therefore costs is group size, with vigilance generally decreasing with increasing group size [4,28,29,30,31,32,33,34,35,36,37]. However, this effect can be levelled off by increasing social vigilance (monitoring of other group members) in larger groups caused by increasing competition and danger of aggression [8,30,38,39,40]. Furthermore, vigilance varies between individuals. Juveniles are often less vigilant than adults [8,35,36,41,42], conspicuous animals are more vigilant [20] and sexes can respond differently to group size, composition and contexts [8,11,14,29,33,38,41,43,44,45,46].

Waterholes are dangerous places as animals visit them on a recurring basis, which attracts predators [30,37,40,47,48,49,50] and requires heightened vigilance. Most studies have been performed on ungulates showing that they are more vigilant when approaching as compared to when drinking [50], particularly under increased hunting or predation pressure [51,52]. This indicates that they assess risks while approaching [50] but then prioritise drinking over vigilance, abandoning any differences in vigilance linked to risks [51]. In contrast, coatis (*Nasua narica*) and white-faced capuchins (*Cebus capucinus*) remained more vigilant while drinking in dangerous situations (below surface waterhole) due to reduced visibility and therefore reduced ability to detect predators [30]. Much less is known about vigilance at waterholes in birds. However, as arboreal animals they differ from terrestrial ones in that they live in three dimensional areas. Across the literature it is assumed that visibility is better when further away from the ground [53,54,55,56], implying that height provides an advantage in detecting predators and that descending to waterholes reduces detectability. Indeed, Votto et al. [57] found that while vegetation cover around waterholes is important for small birds to hide in, they require good visibility to be able to detect predators early, resulting in fewer birds at waterholes with dense vegetation cover. Additionally, the ability to escape is likely to be greater when away from the ground as acceleration is higher for horizontal flight as compared to ascending flight [58]. This has been supported by studies on flight initiation distance, which was higher the closer the birds were to the ground [53,54,55] (see [59]). However, predation pressure can vary between species depending on the main predator type; arial predators pose a greater threat when birds perch higher up in trees, whereas terrestrial predators are more dangerous when birds are lower to the ground [53,55]. Hernandez [56] stated that the better predator detection might outweigh heightened predation pressure when higher up in trees. Taking this all together, one might expect higher vigilance the closer the birds are to the ground due to reduced visibility and ability to flee [58]. What is known about vigilance at waterholes is that birds are more vigilant when they perch in trees without leaves where they are more exposed as compared to trees with leaves, indicating that perceived higher risk of predation increased vigilance [24,60]. Furthermore, vigilance in trees was higher around small waterholes because firstly, predators can focus their attack on a smaller area, which therefore increases predation risk, and secondly, there is potentially higher competition to find a space to drink water, resulting in higher social vigilance [24,60]. Additionally, vigilance is reduced in larger groups across taxa [24,30,50,60,61] but can increase when other groups are present [40].

The current study aimed to investigate vigilance in the colour polymorphic Gouldian finch (*Chloebia gouldiae*) when on the ground while drinking. Gouldian finches are endemic to North Australia, inhabiting tropical savannah grassland [62]. They are nomadic during the non-breeding season [62], tracking the availability of sorghum grasses which is their main food [63]. They fly between two to four km to water sources (creeks, springs) [64], which progressively decrease and disappear over the dry, non-breeding season, resulting in aggregation of larger groups. Gouldian finches drink by dipping their beak in the water and sucking the fluid in, which lasts several seconds. During this time their head is lowered and vigilance is impaired. Common predators around waterholes are Brown falcons (*Falco berigora*) and Brown goshawks (*Accipiter fasciatus*), with the former primarily hunting birds on the ground (ambush or fast flight attack) and the latter hunting birds in trees and on the ground (ambush or fast flight attack) [65]. These two predators made up the majority of attacks on the Gouldian finches. Earlier research has shown that Gouldian finches landing in trees near waterholes have higher vigilance when they perch in leafless trees as compared to trees with leaves providing cover [24]. Additionally, vigilance was higher when perched in trees at small waterholes (higher predation risk, more competition) and when alone [24]. The species occurs in three head-colour morphs, with 70% in the population being black-headed birds, 30% red-headed birds and <1% yellow-headed birds across both sexes [66]. Males have a brighter plumage and a longer central tail feather than females, whereas juveniles are uniformly grey–green [64]. Gouldian finches are listed as endangered by the Australian Department of Environmental Parks, and Water Security [67]. The current study used two measures of vigilance: the frequency of head movements as it allows us to learn about the vigilance strategies used [23], and interscan interval, in order to see whether both variables measure the same or different aspects of vigilance. We predicted the following:

(1) The frequency of head movements increases the closer the birds are to the ground as they become more vulnerable to predation [58].
(2) The frequency of head movements decreases with increasing waterhole size and group size (decreasing predation risk [24]), also considering age and colour morph (conspicuousness [20,60]) and sex as these variables had an effect in other species, e.g., ref. [68].
(3) Interscan interval increases with increasing waterhole size and group size (decreasing predation risk [24]), again considering age, colour morph and sex.
(4) Frequency of head movements and interscan intervals are negatively correlated, reflecting higher vigilance in more dangerous situations [2,17,19,20].

## 2. Materials and Methods

### 2.1. Study Location

Data of Gouldian finches’ vigilance were collected during the dry, non-breeding season between July and September 2023 and 2024 in the Kimberley region in Western Australia. During this period, birds start to gather around the remaining waterholes. Overall, nine waterholes were sampled between Wyndham in the West (15°29′08.3″ S 128°07′14.9″ E), Lake Argyle in the East (16°05′55.4″ S 128°42′17.1″ E) and El Questro Resort in the Southeast (16°00′28″ S 127°58′50″ E). The habitat around all locations was similar and dominated by annual sorghum grass (*Sorghum *spp.) and a mixture of eucalyptus (*Eucalyptus *spp.), bloodwood (*Corymbia *spp.) and boab (*Adansonia gregorii*) trees typical for the species [69]. Waterholes were on median 11.4 km (quartiles 3.3–32.6 km) apart and differed in size from 1 m^2^ (small) to creeks of 100 m length (large) with medium waterholes in between (1.5–5 m^2^; Table 1). The waterholes that were only around 1 km apart were visited by different populations (different group sizes and composition; locations 1 and 2 and 4 and 5) and/or were separated by a ridge (locations 4 and 5). Two locations were sampled in both years (location 1 refers to the same creek but the waterholes were about 50 m apart between years and more surrounded by vegetation in 2024). It is assumed that different birds were sampled in the two years as turnover is high [63]. Predation attempts were common around all waterholes, with daily attacks by Brown falcons and Brown goshawks confirming that waterholes are dangerous places.

### 2.2. Data Collection

Each waterhole was visited two to seven times, depending on the number of birds visiting the waterhole and the sampling method (direct observation or video, see below). Repeated visits were 2.6 days ± 1.1 days apart. Data collection occurred between 5:30 am and 10:00 am. As Gouldian finches visit waterholes once a day [70], it was assumed that different birds were sampled during that period and pseudoreplication was unlikely to occur.

In both years, the observer was positioned about 10–15 m away from the waterhole hidden in vegetation. Birds were observed on the ground through a binocular and data recorded with a Dictaphone. In 2024, additional data were recorded with an action camera (Akaso, Brave 7) positioned 1 m away from the waterhole hidden under leaves or rocks (Table 1). When both methods were used at the same waterhole, Dictaphone data came from an area not covered by the camera, i.e., different birds were sampled. Video recording was restricted to two days per site as many more birds could be sampled when compared to the direct observation method. Data collection started as soon as a Gouldian finch landed on the ground. The head colour, sex and age of the focal bird and all other Gouldian finches on the ground and their numbers were recorded on the Dictaphone before every movement of the head was counted before drinking, during drinking when raising the head and after drinking before flying off by quietly talking into the Dictaphone. Additionally, it was recorded when the bird was drinking allowing the calculation of interscan intervals (see below). The next bird landing was then selected as the new focal bird.

### 2.3. Data Analysis

Data were analysed with IBM SPSS Statistics v 29.0.1.0. All data are available in Table S1. For analysis, the frequency of head movements was calculated as a measure of vigilance by dividing the number of head movements of each individual by the time it took to make these head movements. A higher frequency reflects higher vigilance as the bird changes direction more often in a given time [23].

To address the first prediction that birds become more vigilant as their proximity to the ground increases, the current data were compared with vigilance data collected a year earlier when birds were observed sitting in the trees surrounding the waterholes and landing on perches close to the water [24]. Those data had been collected in the same way as described above. Perches close to the ground were about 30–50 cm above the ground and near the water source. The frequency of head movements was LG_10_ transformed to achieve normality and ANOVA used to compare the dependent variable against the three different levels. Sample sizes were *n* = 1153. Tamhane test was used for post hoc tests as variances were unequal. As some birds might have been sampled repeatedly (e.g., in trees and on the perch close to the ground), bootstrapping was used stratified by location with 10,000 samples. Bootstrapping does not require strong distributional assumptions and uses resampling with replacement to calculate distributional values [71].

To investigate whether social and environmental factors affected vigilance on the ground (prediction 2), Generalised Linear Models (GLM) were used. The dependent variable, frequency of head movements on the ground (*n* = 648), was square root transformed to achieve normally distributed data. For the GLM, an identity link function was used. Fixed effects were waterhole size (3 levels: small: <1 m^2^, *n* = 309; medium: 1–5 m^2^, *n* = 154; large: >10 m^2^, *n* = 183), group size (5 levels: 1: alone, *n* = 250; 2: two birds, *n* = 134; 3: 3–4 birds, *n* = 115; 4: 5–7 birds, *n* = 85; 5: >7 birds, *n* = 62), morph (3 levels: 1: black-headed, *n* = 266; 2: red-headed, *n* = 107; 3: unknown (juvenile), *n* = 273) and sex (3 levels: 1: males, *n* = 241; 2: females, *n* = 132; 3: unknown (juvenile), *n* = 273). For group sizes, the same categorisation was used as in earlier papers [24,60]. Two-way interactions were water size x group size, water size x morph, group size x morph and morph x sex. Year was included as a covariate to account for potentially different conditions between years. Non-significant variables were removed one-by-one, starting with the least significant variable in interaction terms and then the main variables. The omnibus test (Likelihood Ratio chi-square test) was used to validate the final model by comparing the fitted model against the intercept only model [72]. The final model was run with bootstrapping stratified by location with 10,000 samples. For parameter estimates, Wald chi-square statistics was used.

In a third analysis, the interscan interval between vigilance bouts was analysed in relation to the same environmental and social factors as above (prediction 3). The interscan interval was calculated as the mean duration across all occurrences an individual had its heads down while drinking (*n* = 497). The variable was LG_10_ transformed to achieve normality and an identity link function used for the GLM. Year was again used as a covariate and bootstrapping stratified by location with 10,000 samples applied to the final model to account for any double sampling. Wald chi-square statistics were used for parameter estimates.

Lastly, it was investigated whether the two measures of vigilance—frequency of head movements and interscan interval—were correlated on the individual level (prediction 4). Spearman correlations were used (*n* = 497) as transformations did not result in normally distributed data. Bootstrapping was applied stratified by location with 10,000 samples.

## 3. Results

**Figure 1:**
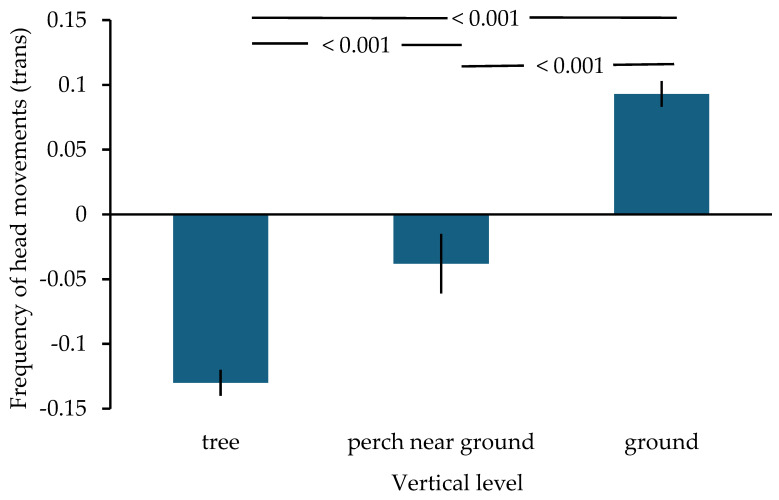
Mean ± SE frequency of head movements in Gouldian finches at tree level, close to the ground and on the ground at waterholes. On the y-axis, zero refers to one head movement/s; positive values = more than one head movement per s; negative values = fewer than one head movement per s. numbers above the bars represent *p*-values derived from post hoc tests (see text).

**Figure 2:**
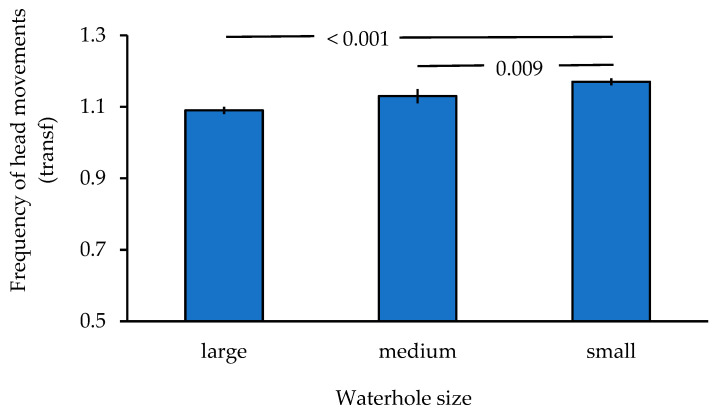
Mean ± SE frequency of head movements in relation to waterhole size. Numbers above the bars reflect *p*-values derived from parameter estimates (see text).

Addressing the first prediction, the frequency of head movements increased the closer the birds came to the ground (ANOVA: *n* = 1153, df = 2, F = 210.567, *p* < 0.001; Table 2), explaining 0.267 of the variance. Post hoc tests showed that the frequency of head movements of birds perched in the tree was significantly lower than of those sitting on a perch near the ground (Tamhane: *p* < 0.001) or when on the ground (*p* < 0.001). Additionally, there was a lower frequency of head movements when sitting on a perch near the ground as compared to being on the ground (*p* < 0.001; Figure 1). Bootstrapping resulted in the same outcome. Once on the ground (prediction 2), the frequency of head movements was affected by waterhole size alone (Table 3) with lower frequencies at both, medium (GLM parameter estimate Wald Chi^2^: *n* = 648, Chi^2^ = 5.637, *p* = 0.018 (*p* = 0.009 with bootstrapping)) and large waterholes Chi^2^ = 27.349, *p* < 0.001) as compared to small waterholes (Figure 2). No other variable had an effect. This model was significantly better than the intercept-only model (Omnibus test Chi^2^ = 338.302, df = 3, *p* < 0.001).

**Figure 3:**
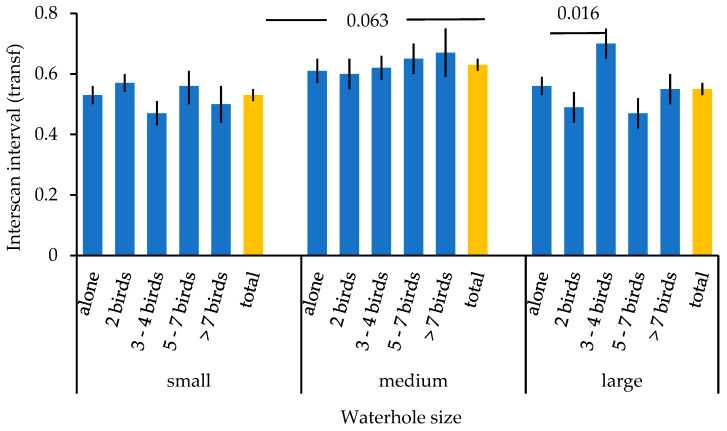
Mean ± SE interscan interval in relation to waterhole size and group size. The yellow bars provide the overall mean ± SE interscan interval for each waterhole size. Values above the bars represent *p*-values derived from parameter estimates (see text).

**Figure 4:**
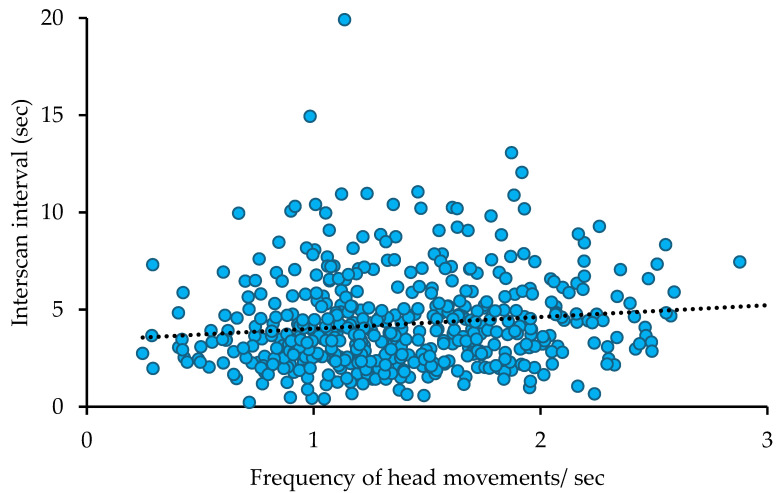
Correlation between frequency of head movements and interscan interval. The dotted line is the trend line.

The interscan interval when drinking (prediction 3) was linked to waterhole size and the interaction term waterhole size x group size (Table 4). Birds at medium-sized waterholes showed a trend for longer interscan intervals as compared to birds at small waterholes (GLM parameter estimates Wald Chi^2^: *n* = 497, Chi^2^ = 2.655, *p* = 0.103 (*p* = 0.063 with bootstrapping)), whereas no significant differences were found between birds at large and small waterholes Chi^2^ = 0.506, *p* = 0.477; Figure 3). From the interaction term, only group sizes of 3–4 birds at large waterholes had significantly longer interscan intervals as compared to single individuals at large waterholes (Chi^2^ = 5.232, *p* = 0.22 (*p* = 0.016 with bootstrapping), Figure 3). Differences seem to be more linked to the direction of changes in interscan interval with increasing group sizes. As reported at large waterholes, the longest interscan interval by far was found in group sizes of 3–4 birds, whereas smaller or larger group sizes had shorter interscan intervals (Figure 3). In contrast, interscan intervals steadily increased with group size at medium-sized waterholes, whereas no pattern was found at small waterholes (Figure 3). No other variables had a significant effect. This model was significantly better than the intercept only model (Omnibus test: Chi^2^ = 52.505, df = 15, *p* < 0.001). Finally, at the individual level, the frequency of head movements was positively correlated with the interscan interval when drinking (prediction 4; Spearman correlation: *n* = 497, r = 0.156, *p* < 0.001; Figure 4).

## 4. Discussion

Two measures of vigilance, the frequency of head movements and interscan interval, were investigated in Gouldian finches at waterholes while they were drinking at waterholes. Gouldian finches increased the frequency of head movements, indicating increased vigilance, the closer they were to the ground. When on the ground, the frequency of head movements between drinking bouts and, therefore, vigilance, was higher at small waterholes compared to larger waterholes. Both results can be linked to higher perceived predation risk. Additionally, interscan interval varied with waterhole size and group size in a way that likely reflects a combination of antipredator behaviour and social vigilance. Finally, interscan interval was positively correlated with frequency of head movements, which was surprising.

The first prediction stated that the frequency of head movements should increase the closer the birds were to the ground. This was indeed the case, indicating that the birds perceived descending to the ground as becoming more vulnerable to predation. This might be due to being less able to spot approaching predators [53,54,55,56] and/or being slower at take-off from the ground when compared to perching in a tree [58]. Heightened vigilance in situations that were perceived as more dangerous has been found in other studies [9,14,15]. In ungulates, vigilance is often higher during approach than during drinking [50,51,52]. However, these studies have not investigated whether vigilance changes during the approach, e.g., when leaving cover and when in increasing proximity to the water source.

The frequency of head movements during drinking varied with waterhole size. Birds showed a higher frequency of head movements (higher vigilance) at small waterholes as compared to medium and large waterholes confirming the second prediction. This is consistent with higher vigilance when perching in trees around small waterholes found in Gouldian finches and Long-tailed finches (*Poephila acuticauda*; refs. [24,60]). Small waterholes are more dangerous as predators can focus their attack on a smaller area. Additionally, there can be more competition about places to drink at small waterholes [30,37]. Both factors increase vigilance concurrently. Higher vigilance while drinking at waterholes that were more dangerous was also found in coati and white-faced capuchins [30]. However, the results contrast with studies on ungulates reporting that vigilance differences disappeared once drinking [51]. Higher vigilance at small waterholes becomes a recurring theme and encompasses the entire time around waterholes from perching in the tree [24] to being on the ground and is possibly more widespread than expected [60]. This causes concern as environmental temperatures and the frequency of heatwaves are forecasted to increase in the future [73], resulting in small waterholes occurring earlier in the dry season and for longer periods of time. When increased vigilance at waterholes is linked to stress [74], then birds will be exposed to such stressful events over longer time periods, which can be detrimental to their overall health. This is an area that requires more research.

The frequency of head movements during drinking was not affected by group size, which is surprising and is in contrast to our prediction. Earlier studies found that Gouldian finches have higher vigilance when perched alone in a tree around waterholes as compared to being with others [24]. However, the size of the group had no effect on vigilance. Similar results were found in Long-tailed finches perching in trees at waterholes [60]. Furthermore, group size affected vigilance during drinking in a range of species with lower vigilance in larger groups [30,50,61]. Group sizes changed quickly while birds were drinking as other Gouldian finches often dropped down to the water once a Gouldian finch had made a start. Therefore, the recorded group sizes might have been inaccurate. However, group sizes affected interscan intervals, making this explanation unlikely. Alternatively, Gouldian finches were rarely entirely alone as they usually follow other Estrildid finches, e.g., Long-tailed finches, to the ground [75]. While the presence of other finch species did not affect Gouldian finch’s vigilance when sitting in the tree [24], it might have affected them when on the ground, possibly because birds are closer together providing more protection against predators [76]. Future studies should also consider the presence of other species.

Complicating patterns were found for interscan interval (third prediction). There was a trend for longer interscan intervals at medium waterholes as compared to small ones, which was expected when small waterholes are perceived as more dangerous [2,16,17]. Looking up at shorter intervals increases the chance of detecting predators [16]. However, no differences in interscan interval were found between large and small waterholes. Possibly, interaction with group size masked effects of waterhole size. The longest interscan interval was indeed found at large waterholes with three to four Gouldian finches present and was significantly higher than when drinking alone. The combination of waterhole size and moderate group size seemed to be perceived as the safest situation. Smaller numbers increase the risk of being targeted (safety in numbers; ref. [74]), whereas larger numbers might require more social vigilance [8,30,38,39,40]. Additionally, birds might be more spread out at larger waterholes, reducing the group effect. Groups of three to four birds might be families that drink closer together. At medium-sized waterholes, interscan intervals became continually longer with increasing group size (although not significantly), which is consistent with other studies [30]. The group effect might be more prevalent here as birds are likely closer together than at large waterholes. Finally, no pattern was found in relation to group size at small waterholes. The higher perceived predation at small waterholes might dampen any group effects and/or competition might counteract any group effects [30,37].

Frequency of head movements and interscan interval were positively correlated with individuals having a higher frequency of head movements, showing a longer interscan interval. This is somehow surprising and in contrast to our fourth prediction as a higher frequency of head movements reflects higher vigilance [24,25,26], whereas a longer interscan interval is generally associated with lower vigilance [2,17]. Additionally, higher vigilance (higher vigilance rate) was accompanied by shorter interscan intervals in Siskins [19,20] and chaffinches (*Fringilla coelebs*; ref. [16]). In the Pascual and Senar [19] study, the higher vigilance rate and shorter interscan interval were linked to a proactive personality type, resulting in overall higher vigilance when compared to reactive siskins. Perhaps frequency of head movements and interscan interval constitute two different strategies for responding to a threat. These different strategies do not necessarily have to be linked to personality traits but could be used in different contexts. For example, one strategy might centre around the frequency of head movements with fast scans in all directions (high vigilance) between drinking bouts. The high frequency of head movements allows better detection of threats [25]. When the environment is deemed safe, drinking is prioritised with long drinking bouts. The other strategy might centre more around the interscan interval. While an individual might just raise its head and look in one direction for longer (lower vigilance), this is counteracted by shorter interscan intervals, i.e., shorter drinking bouts, potentially compromising drinking to some extent. Shorter interscan intervals have been linked to higher vigilance [2,17] and situations that are more threatening [16,19,20]. Shorter interscan intervals have also been associated with higher vigilance rates [16,19,20], which have been linked to higher predator detection [16]. Potentially, shorter interscan intervals also increase predator detection. Therefore, it is suggested that both, an increased frequency of head movements or a shorter interscan interval, can be used to scan for threats successfully. Which strategy is used might be linked to the state of the individual. For example, thirsty individuals might use a higher frequency of head movements to have longer drinking bouts. Alternatively, individuals could choose to look for longer in one direction with shorter interscan intervals to track other individuals [23]. Indeed, interscan interval was affected by group size, although in a complicating manner in interaction with waterhole size. More research is required regarding whether the frequency of head movements and interscan interval constitute different strategies and whether these are linked to personality traits or vary with context as suggested here.

## 5. Conclusions

In conclusion, vigilance measured as the frequency of head movements increased as the birds’ proximity to the ground increased, possibly as a consequence of reduced sight and increased difficulty to take off. The frequency of head movements during drinking was high and affected by waterhole size with a higher frequency at small waterholes reflecting higher vigilance. With more prolonged dry seasons, birds may become increasingly vigilant, which if linked to stress can be detrimental and requires further attention. Interscan interval showed a trend to be longer at medium-sized waterholes as compared to small waterholes, reflecting increased perception of safety but interscan intervals at large waterholes were similar to those at small waterholes. Group size interactions along with waterhole size affected interscan intervals, showing situation-dependent adjustments in vigilance. Finally, the frequency of head movements was positively correlated with interscan interval, potentially representing two different scanning strategies, which requires further investigation.

## Figures and Tables

**Table 1:** Waterhole locations and sampled birds.

	Distance Between Adjacent Sites (km)	Waterhole Size	Recording Method	Number of Birds Sampled 2023	Number of Birds Sampled 2024
1	1.0 *	small	D (2023) & V (2024)	142	128 **
2	11.1	medium	D & V		94
3	13.8	large	D		58
4	1.5	small	D	30	
5	8.6	medium	D	26	
6	38.8	large	D	7	
7	11.7	large	D & V	21	98
8	80.6	medium	D		34
9		small	D	10	

* Site 1 is 1 km from site 2; ** the waterhole in 2024 was about 50 m further down the creek and more secluded than in 2023; small waterholes were <1 m^2^, medium waterholes ranged from 1.5 to −5 m^2^ and large waterholes were >5 m^2^ in size. D: Dictaphone, V: Video.

**Table 2:** ANOVA output for frequency of vigilance in relation to vertical level.

Variable	df	F-Value	*p*-Value
Corrected model	2	210.567	<0.001
Intercept	1	12.331	<0.001
Vertical level	2	210.567	<0.001

**Table 3:** Final GLM output for frequency of vigilance in relation to environmental and social factors.

Variable	df	Chi-Square	*p*-Value
Intercept	1	989.105	<0.001
Waterhole size	2	27.515	<0.001
Year	1	425.567	<0.001

Year was used as a covariate.

**Table 4:** Final GLM output for interscan intervals in relation to environmental and social factors.

Variable	Df	Chi-Square	*p*-Value
Intercept	1	99.738	<0.001
Waterhole size	2	12.398	0.002
Group size	4	1.788	0.775
Waterhole size x group size	8	18.198	0.020
Year	1	8.960	0.003

Year was used as a covariate.

## Data Availability

All data are available in the Supplementary Materials section of this paper.

## Supplementary Materials

The following supporting information can be downloaded at https://www.mdpi.com/article/10.3390/ani16010087/s1, Table S1: Data file.
